# Genome-Wide Investigation and Functional Analysis Reveal That *CsGeBP4* Is Required for Tea Plant Trichome Formation

**DOI:** 10.3390/ijms24065207

**Published:** 2023-03-08

**Authors:** Hao Zhou, Wei Zhou, Xinzhuan Yao, Qi Zhao, Litang Lu

**Affiliations:** 1 College of Tea Sciences, Institute of Plant Health & Medicine, Guizhou University, Guiyang 550025, China; 2 The Key Laboratory of Plant Resources Conservation and Germplasm Innovation in Mountainous Region (Ministry of Education), Institute of Agro-Bioengineering/College of Life Sciences, Guizhou University, Guiyang 550025, China

**Keywords:** *Camellia sinensis*, trichome formation, *CsGeBP*, transcriptional regulation

## Abstract

Tea plant trichomes not only contribute to the unique flavor and high quality of tea products but also provide physical and biochemical defenses for tea plants. Transcription factors play crucial roles in regulating plant trichome formation. However, limited information about the regulatory mechanism of transcription factors underlying tea plant trichome formation is available. Here, the investigation of trichome phenotypes among 108 cultivars of Yunwu Tribute Tea, integrated with a transcriptomics analysis of both hairy and hairless cultivars, revealed the potential involvement of *CsGeBPs* in tea trichome formation. In total, six *CsGeBPs* were identified from the tea plant genome, and their phylogenetic relationships, as well as the structural features of the genes and proteins, were analyzed to further understand their biological functions. The expression analysis of *CsGeBPs* in different tissues and in response to environmental stresses indicated their potential roles in regulating tea plant development and defense. Moreover, the expression level of *CsGeBP4* was closely associated with a high-density trichome phenotype. The silencing of *CsGeBP4* via the newly developed virus-induced gene silencing strategy in tea plants inhibited trichome formation, indicating that *CsGeBP4* was required for this process. Our results shed light on the molecular regulatory mechanisms of tea trichome formation and provide new candidate target genes for further research. This should lead to an improvement in tea flavor and quality and help in breeding stress-tolerant tea plant cultivars.

## 1. Introduction

The tea plant (*Camellia sinensis* (L.) O. Kuntze) is one of the most popular nonalcoholic beverage crops worldwide. Tea trichomes, also referred to as ‘Cha Hao’, greatly contribute to the unique flavor and health benefits of tea products since they are rich in secondary metabolites, such as catechins, theanine, caffeine, flavonols, and various volatiles [1]. Generally, the distribution of tea trichomes is associated with the degree of tissue tenderness and varies significantly among different tea plant varieties and cultivars [2,3,4,5]. Apical buds and young leaves are the main materials processed for tea products and are usually enriched with trichomes, which is an important feature associated with superior tea quality for many brands of commodity teas, such as white teas, high-quality black teas, and green teas [1,6]. Therefore, trichome density is considered one of the most important criteria for tea quality evaluation. Moreover, tea plant trichomes also act as protective barriers against abiotic and biotic stresses via divergent strategies, such as the reflection of ultraviolet and high lights, reduction in water loss under high temperatures, prevention of leaf freezing in response to cold stress, and resistance to pathogen and herbivore attacks [1,6,7,8,9,10,11,12]. Due to the importance of trichomes for both tea quality and defense, insights into the molecular bases of trichome initiation, growth, and development are needed to improve tea quality and the breeding of stress-resistant tea cultivars.

The formation of plant trichomes is regulated by numerous factors, including environmental cues, hormones, and regulatory genes [13]. Among them, a series of transcription factors playing vital regulatory roles in trichome initialization, growth, and development have been identified in a number of plant species, such as *Arabidopsis* [4,14,15,16,17,18], cucumber (*Cucumis sativus*) [19], tomato (*Solanum lycopersicum*) [20], *Brassica campestris* [21] and *B. napus* [22], and the functions of some genes have been explored in depth. Among these, positive regulatory factors have been proven to enhance the development of trichomes by forming an MYB-bHLH-WD40 protein (MBW) transcriptional complex and subsequently activating the downstream homeodomain–leucine zipper (HD-Zip) transcription factor *GLABRA2* (*GL2*) and other effectors [7,23,24,25]. The components of the MBW complex are functionally redundant and mainly include (i) R2R3 MYB-related transcription factors, GLABRA1 (GL1), MYB23, and MYB5; (ii) bHLH-like transcription factors, GLABRA3 (GL3), ENHANCER OF GLABRA3 (EGL3), TRANSPARENT TESTA 8 (TT8), and MYC-1; and (iii) a WD40-repeat protein, TRANSPARENT TESTA GLABRA1 (TTG1) [13,25,26,27,28,29,30,31,32,33,34]. Moreover, SAD2 was also identified as a positive regulator due to its function in maintaining the stability of the MBW complex [35]. In addition, CAPRICE (CPC), TRIPTY CHON (TRY), ENHANCER OF TRY AND CPC1 (ETC1), and ETC2 were identified as negative regulators of the formation of trichomes via interactions with GL3, EGL3, and TTG1 [13,36].

Tea trichomes are differentiated from epidermal cells and belong to the unicellular, unbranched, and non-glandular type of surface hairs, which are similar to those of *Arabidopsis* [1,37]. However, unlike the model plant *Arabidopsis*, the lack of a stable transgenic system in tea plants constrained the progress of studies on the transcription-factor-regulated mechanisms of tea trichome formation conducted in recent decades. Until recently, an R2R3 MYB transcription factor CsMYB1 was confirmed to regulate trichome formation in tea plants by forming an MBW complex with CsGL3 and CsWD40, thereby activating the trichome regulator genes *CsGL2* and *CsCPC*, and the galloylated *cis*-catechins biosynthesis genes *anthocyanidin reductase* and *serine carboxypeptidase-like 1A* [4,38]. In addition, comparative transcriptomics research was conducted between hairless ‘Chuyeqi’ (CYQ) and hairy ‘Budiaomao’ (BDM) tea plants, and a total of 208 transcription factors were differentially expressed [5]. Among them, *CsMYB75*, *CsNOK*, and *CsATML1* might enhance trichome development by up-regulating their expression levels, while *CsSPL6* and *CsSPL12* were regarded as negative regulators in trichome formation due to their higher expression levels in the hairless cultivar ‘CYQ’ than those in the hairy ‘BDM’ [5]. Another comparative transcriptomic study was carried out between the hairless ‘Rongchunzao’ and hairy ‘Fudingdabai’ tea plant cultivars, which provided several candidate regulatory transcription factors associated with trichome development, including gene members of the *HD-Zip*, *ZEP*, *SPL*, *MADS-box*, *TCP*, *and GRF* families [2]. Additionally, several transcription factor gene families, such as *bHLHs* and *CPC-like* genes, were identified from the tea plant genomes and were thought to play roles in tea trichome formation [3,39]. However, the specific mechanisms associated with tea trichome formation regulated by genes identified using comparative transcriptomics and genome-wide research are still elusive. Recently, virus-induced gene silencing (VIGS) technology has been successfully established in tea plants, enabling the efficient and accurate analysis of gene functions in tea plants [40]. Mutants can be easily obtained without genetic transformation in tea plants using VIGS technology, and the resulting silencing of targeted genes can be maintained for a relatively long time. This newly developed VIGS technology introduces a new method of conducting functional studies on regulatory genes involved in tea plant trichome formation.

The glabrous-enhancer-binding protein (GeBP) family members are plant-specific transcription factors whose members share a central DNA-binding domain. They were proven to play critical regulatory roles in cell expansion in *Arabidopsis* [41]. Another study revealed that AtGeBP interacted with the trichome-formation-related gene *AtGL1* in yeast and in vitro, and its expression was regulated by *KNAT1*, a meristem establishment gene [42,43]. These findings suggest that *AtGeBP* might participate in plant trichome formation. In addition, it was reported that GeBP family genes were involved in plant responses to phytohormones such as gibberellin [18,42,44,45], cytokinin [45,46,47,48], and auxin [45]. Furthermore, their roles in plant responses to environmental stresses such as heavy metals and pathogens were also reported [38,45,49]. For example, *GeBP-like 4* (*GPL4*) was induced in response to cadmium, copper, and zinc stresses in *Arabidopsis* [49]. Thus far, the genome-wide identification and characterization of the *GeBP* gene family have been conducted in soybean [50], mango (*Mangifera indica*) [38], moso bamboo (*Phyllostachys edulis*) [51], tomato (*Solanum lycopersicum*) [52] and nine Gramineae crops (*Brachypodium distachyon*, *Hordeum vulgare*, *Oryza sativa* ssp. *Indica*, *Oryza sativa* ssp. *japonica*, *Oryza rufipogon*, *Sorghum bicolor*, *Setaria italica*, *Triticum aestivum*, *Zea mays*) [45]. However, the specific biological functions of most *GeBPs* have not been characterized. Furthermore, the function and regulatory mechanism of *CsGeBPs* in tea plant trichome formation are also elusive.

In this study, we integrated the investigations of trichome phenotypes and transcriptome profiling of different tea plant cultivars. The *CsGeBP* family members were screened out as candidate regulatory genes regulating tea plant trichome formation. The phylogenetic relationship, gene, protein structural features, and tissue-specific and environmental-responsive expression patterns of *CsGeBP* family members were characterized. The subsequent correlation analysis revealed a close relationship between the gene expressions of *CsGeBP4* and a high-density trichome phenotype, and the indispensable regulatory role of *CsGeBP4* in trichome formation was further verified by the VIGS strategy performed in tea plants, indicating that *GeBP4* is required for regulating tea plant trichome growth and development. This study provides reliable in vivo evidence for the involvement of the *GeBP* family member in plant trichome formation and proposes a new perspective on the potential transcriptional regulation mechanism involving *CsGeBP4* during tea plant trichome formation. This establishes a theoretical framework and valuable foundation for future research to improve tea flavor and quality, as well as to continue to breed stress-tolerant tea plant cultivars.

## 2. Results

### 2.1. Association of Trichome Phenotype with Transcription Factor CsGeBPs

**Figure 1:**
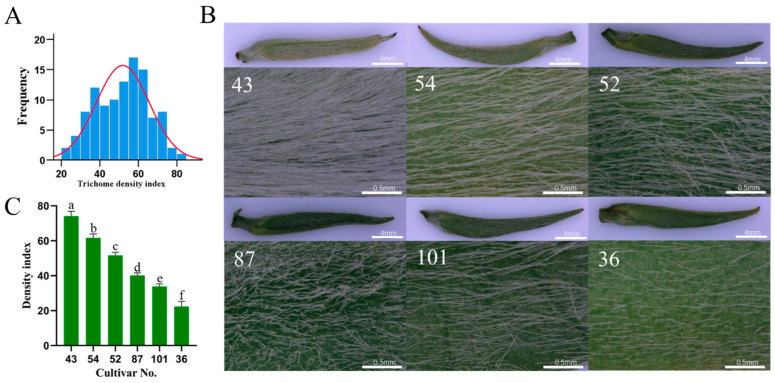
Trichome phenotypes among different tea plant cultivars. (**A**) Normal distribution test of the tea trichome density phenotype among the 108 cultivars of Yunwu Tribute Tea. The normal curves are shown in red. (**B**) Trichome phenotypes in the apical buds of selected tea plant cultivars (No. 43, No.54, No. 52, No. 87, No. 101, and No. 36). (**C**) Trichome density index of the apical buds of selected tea plant cultivars. Lowercase letters indicate significant differences among different samples.

To understand the trichome morphological variations in different tea plant cultivars and the underlying genetic factors regulating trichome formation, we investigated the trichome phenotypes on the apical buds of 108 cultivars of Yunwu Tribute Tea (*Camellia sinensis* (L.) Kuntze var. niaowangensis Q. H. Chen), whose tender leaves are used as raw materials for producing one of the internationally famous teas, Guiding Snow Bud [53]. The trichome density index (TDI), analyzed using ImageJ software (https://imagej.en.softonic.com/, accessed on 15 May 2022), was used to evaluate and quantify the trichome phenotypes in tea plants. TDIs were continuously distributed in 108 tea plant cultivars (Figure 1A). Six tea plant cultivars showing significant variations of TDIs were selected to present typical trichome phenotypes with TDIs ranging from high to low levels among the whole population (Figure 1B,C). The apical buds of cultivar No. 43 were covered with the densest trichomes among the six cultivars, and its TDI was 3.3-fold higher than that of cultivar No. 36, which showed the lowest trichome density compared with the other cultivars (Figure 1B,C).

**Figure 2:**
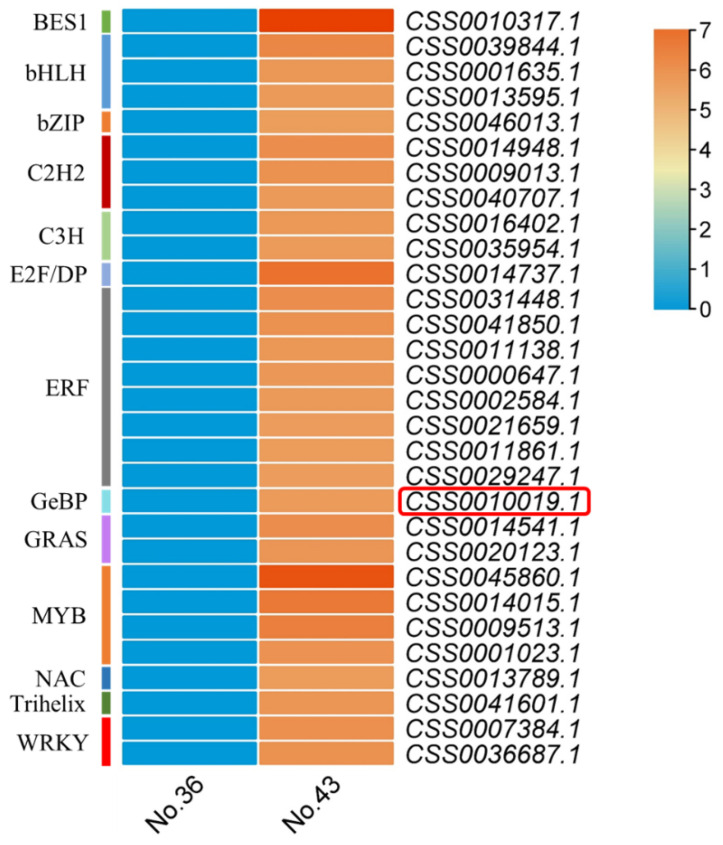
Heatmap of expression changes in putative transcription factors involved in trichome formation. Red indicates up-regulation, and blue indicates genes expressed at background levels. The scale bar represents the log2-based fold changes among two cultivars (hairless No. 36 and hairy No. 43) of Yunwu Tribute Tea. The gene ID of *CsGeBP4* is indicated with a red box.

The RNA-seq transcriptome profiling on apical buds of tea plant cultivars No. 43 and No. 36 was further compared to search for the key regulators affecting trichome formation in tea plants. The top 30 up-regulated transcription factor genes with the highest fold changes in expression levels in the hairy cultivar No. 43 compared with the hairless cultivar No. 36 were extracted from the transcriptome data (Figure 2). These genes belong to 13 gene families, and members from *bHLH*, *bZIP*, *C2H2*, *ERF*, *GRAS*, *MYB*, *NAC*, *Trihelix*, and *WARK* families were identified as differentially expressed genes in previous comparative transcriptome studies conducted between hairy and hairless tea plant varieties [2,4,5]. Among the rest four gene families, only *GeBPs* were reported to be involved in plant trichome formation in previous studies [42,43,50], while their regulatory functions in tea plant trichome formation have not yet been studied. In the present study, a candidate *GeBP* gene (gene ID: CSS0010019.1; *CsGeBP4*) was expressed at higher levels in the hairy cultivar than in the hairless cultivar (Figure 2), indicating that members of the *GeBP* family might also be related to trichome formation in tea plants. Therefore, we further investigated the structural features of genes and proteins, phylogenetic relationships, and expression variations, as well as the functional patterns of *GeBP* family members, aiming to specify their regulatory roles in trichome growth and development in tea plants.

### 2.2. Identification and Phylogenetic Analysis of GeBP Family in Tea Plants

A total of six *GeBP* genes were identified in the tea plant genome and named *CsGeBP1* to *CsGeBP6* based on their chromosomal localization order (Table 1). The biophysical and chemical properties of *GeBPs* are shown in Table 1. The genomic DNA size of *GeBPs* varied from 673 bp (CsGeBP2) to 2624 bp (CsGeBP4). The sequence lengths of GeBP proteins were between 221 (CsGeBP2) and 410 (CsGeBP4) amino acids. Their molecular weights and theoretical isoelectric points varied from 24.6 kDa (CsGeBP2) to 45.0 kDa (CsGeBP4) and 4.63 (CsGeBP3) to 9.85 (CsGeBP2), respectively. Furthermore, all of the CsGeBP members were localized to the nucleus based on the bioinformatics prediction.

**Figure 3:**
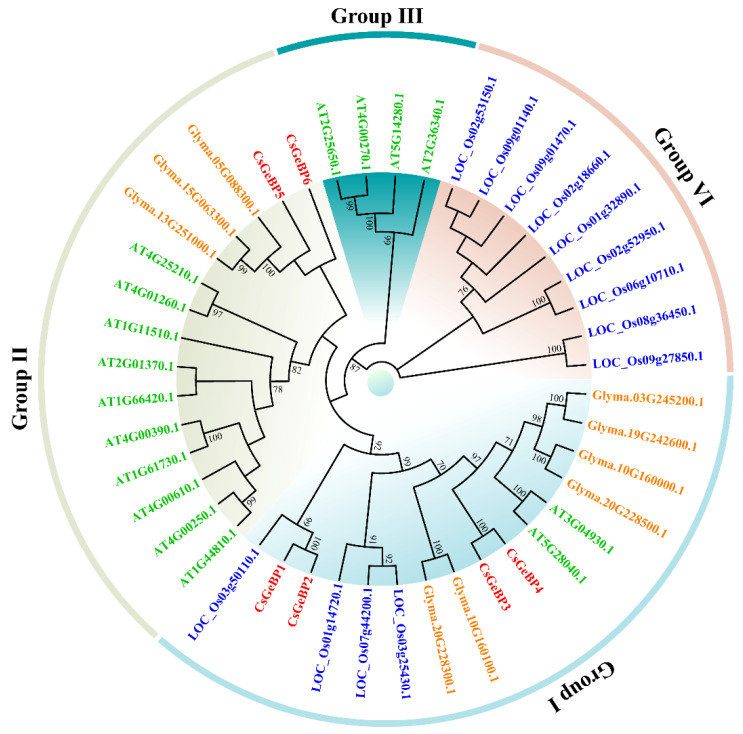
Phylogenetic relationships and classifications of GeBP proteins from tea plants (*Camellia sinensis*), *Arabidopsis thaliana*, rice (*Oryza sativa*), and soybean (*Glycine max*). The phylogenetic tree was constructed via MEGA X, using the neighbor-joining (NJ) method and 1000 bootstrap replicates. All of the GeBPs are divided into four groups. Gene IDs are indicated in different colors representing proteins from different plant species. Red represents tea plants, green represents *Arabidopsis*, blue indicates rice, and orange represents soybean.

To explore the evolutionary relationships of GeBPs in tea plants with their homologs in other plant species, a multi-species phylogenetic tree of GeBPs from tea plants, *Arabidopsis*, rice, and soybean was constructed using the neighbor-joining method (Figure 3; Table S1). A total of 44 GeBPs were divided into four major groups. The six CsGeBPs only appeared in two groups, and close homology was found in three pairs of CsGeBPs (CsGeBP1 and CsGeBP2; CsGeBP3 and CsGeBP4; CsGeBP5 and CsGeBP6). Group I contained the largest numbers of GeBPs, including six GmGeBP, four CsGeBPs, four OsGeBPs, and two AtGeBPs, accounting for 36% of the total GeBPs. Fifteen GeBPs were classified into Group II, with ten, three, and two GeBPs from *Arabidopsis*, soybean, and tea plants, respectively. None of the OsGeBPs were included in this group, indicating that GeBP family members in Group II might be specific to dicots. Group III and IV consisted of four AtGeBPs and nine OsGeBPs, respectively, suggesting that unequal loss and expansion of GeBPs might appear during species differentiation.

### 2.3. Chromosomal Distribution and Gene Structure of CsGeBPs

**Figure 4:**
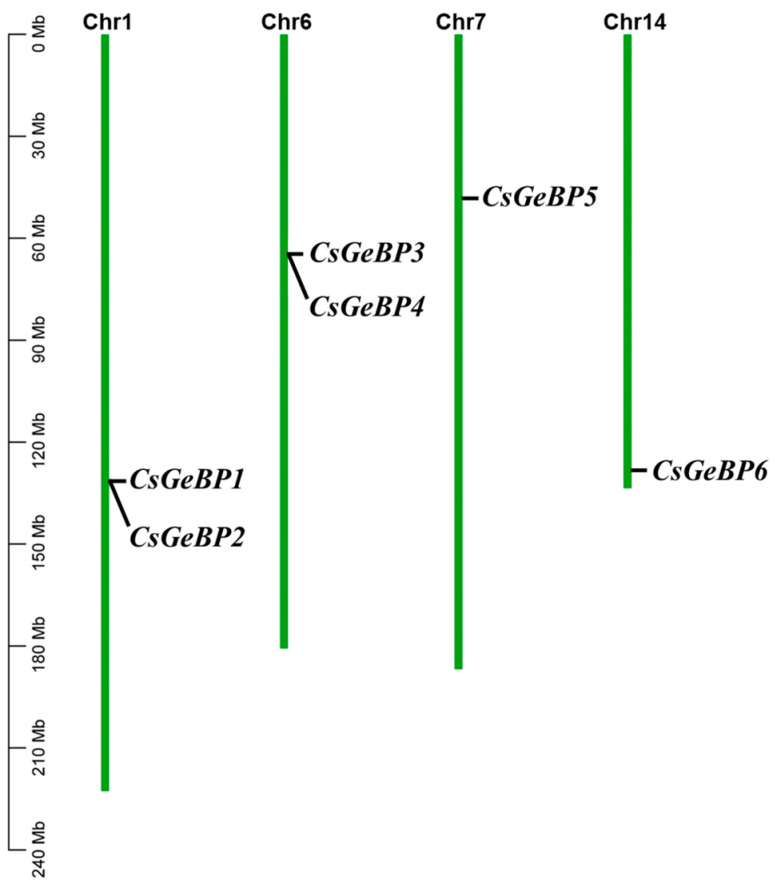
Distribution of *CsGeBP* genes on the tea plant chromosomes. All of the six *CsGeBP* genes were localized to tea chromosomal regions.

The *CsGeBP* gene sequences were mapped onto the tea plant genome to investigate their chromosomal localization. The physical position of each *CsGeBP* gene on the tea plant chromosome is marked in Figure 4. The six *CsGeBP* genes were unevenly distributed on four out of fifteen chromosomes. In detail, *CsGeBP1* and *CsGeBP2* were localized on chromosome 1, while *CsGeBP3* and *CsGeBP4* were distributed on chromosome 6. In addition, *CsGeBP5* and *CsGeBP6* were mapped on chromosomes 7 and 14, respectively. These data provided extra evidence for the analysis of phylogenetic relationships among *CsGeBPs*.

**Figure 5:**
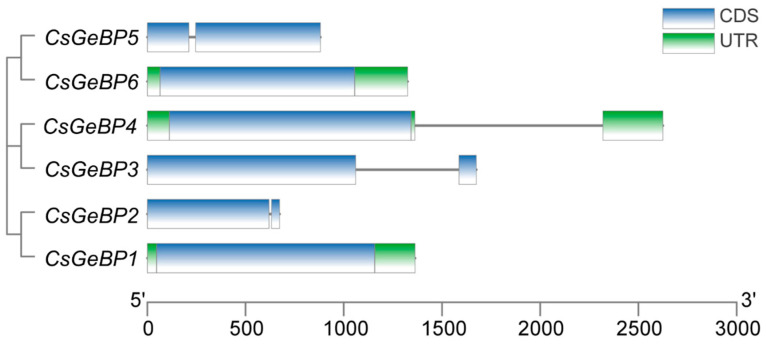
Exon–intron structure of *CsGeBPs*. Green boxes indicate untranslated 5′—and 3′—regions, blue boxes represent exons, and black lines indicate introns.

The analysis of exon–intron organization was performed to understand the gene structural features of the *CsGeBP* family genes (Figure 5). *CsGeBP1*, *CsGeBP4*, and *CsGeBP6* possessed one exon, while *CsGeBP2*, *CsGeBP3*, and *CsGeBP5* contained two exons. Furthermore, *CsGeBP1* and *CsGeBP6* were found to be without introns, and the other four *CsGeBPs* contained only one intron, indicating that the gene structure of *CsGeBPs* might be stable and not prone to being alternatively spliced in the process of gene replication.

### 2.4. Conserved Motif Analysis of the CsGeBP Proteins

**Figure 6:**
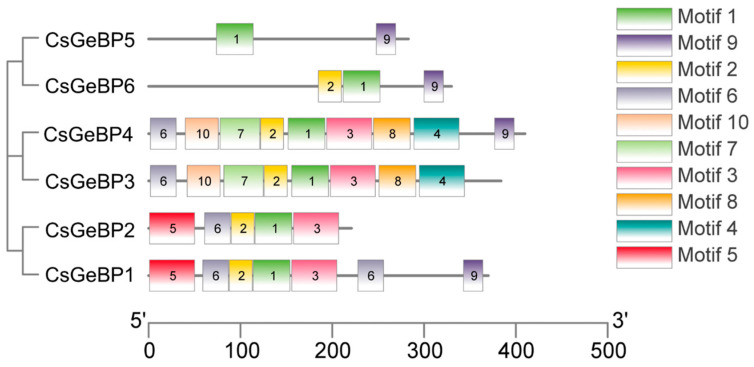
Conserved protein motif analysis of the CsGeBPs. Motifs 1 to 10 displayed by different colors represent different conserved protein motifs. The order of the motifs corresponds to their position within an individual protein sequence.

A total of fifteen distinct motifs were predicted in the CsGeBP family using the MEME program (Figure 6). The number of motifs contained in each CsGeBP ranged from 5 to 10. Both CsGeBP3 and CsGeBP4 contained ten conserved motifs; CsGeBP1 contained nine conserved motifs; and CsGeBP2, CsGeBP5, and CsGeBP6 contained five conserved motifs, respectively. Motif 1 was the most conservative motif due to its presence in all CsGeBPs, and Motifs 2, 3, 6, and 9 were widespread in at least four of the CsGeBPs, indicating these motifs were conserved during the evolution of gene families. CsGeBP3 and CsGeBP4 shared nine common motifs, and CsGeBP1 and CsGeBP2 contained five common motifs, which was highly consistent with their close phylogenetic relationships.

### 2.5. Cis-Regulatory Elements: Analysis of CsGeBP Gene Promoters

**Figure 7:**
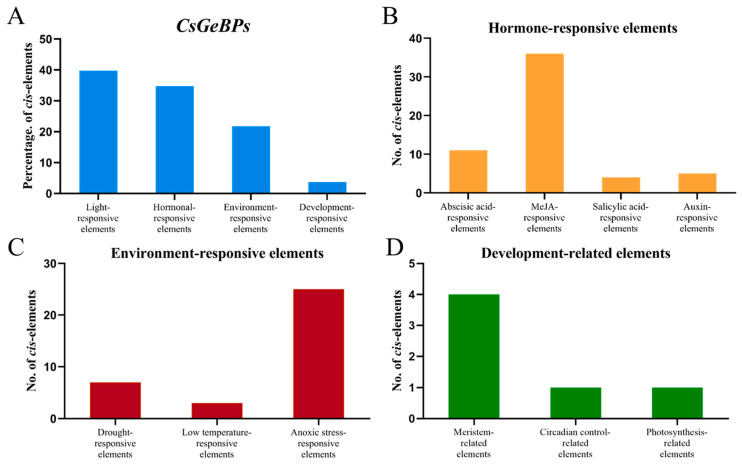
Analysis of the *cis*-regulatory elements in the promoter regions of *CsGeBP* genes. (**A**) The percentage of light-responsive elements, hormone-responsive elements, environment-responsive elements, and plant-growth-related elements in all *CsGeBP* family members. (**B**) Different hormone (ABA, MeJA, auxin, salicylic acid)-responsive elements in the *cis*-element regions of *CsGeBP* genes. (**C**) Different environmental stress (drought, low temperature, and anoxic stress)-responsive elements in *cis*-element regions of *CsGeBP* genes. (**D**) Different plant development-related elements in *cis*-element regions of *CsGeBP* genes.

The modular composition of *cis*-regulatory elements in gene promoter regions plays key roles in regulating gene expression patterns in response to both internal signals and environmental factors. The promoter sequences of *CsGeBP* genes were analyzed to identify *cis*-regulatory elements (Figure 7; Table S2). A total of 161 *cis*-regulatory elements representing 29 non-redundant elements were found in the promoter regions of *CsGeBP* genes, and they were divided into four main groups: light response (39.8%), stress response (34.8%), hormone response (21.7%) and plant developmental regulation (3.7%) (Figure 7A). In the hormone-responsive group, elements associated with MeJA response were highly enriched in *CsGeBP* promoters, followed by ABA- and auxin-responsive elements (Figure 7B). Among all the environmental factors, light might be the most influential factor in the expression of *CsGeBPs*, followed by anoxic stress (Figure 7A,C). In addition, three elements involved in meristem expression, circadian control, and photosynthesis, respectively, were also found in *CsGeBP* promoters (Figure 7D). These results were consistent with the multiple roles of *GeBPs* in plant responses to phytohormones and environmental factors, as well as in plant growth reported by previous studies [38,41,42,43,45,46,47,48,49]. Furthermore, the composition modes of *cis*-regulatory elements were similar between *CsGeBP1* and *CsGeBP2*, as well as *CsGeBP3* and *CsGeBP4*, respectively, which was consistent with their corresponding phylogenetic relationships (Table S2).

### 2.6. Expression Pattern Analysis of CsGeBP Gene in Tea Plants

**Figure 8:**
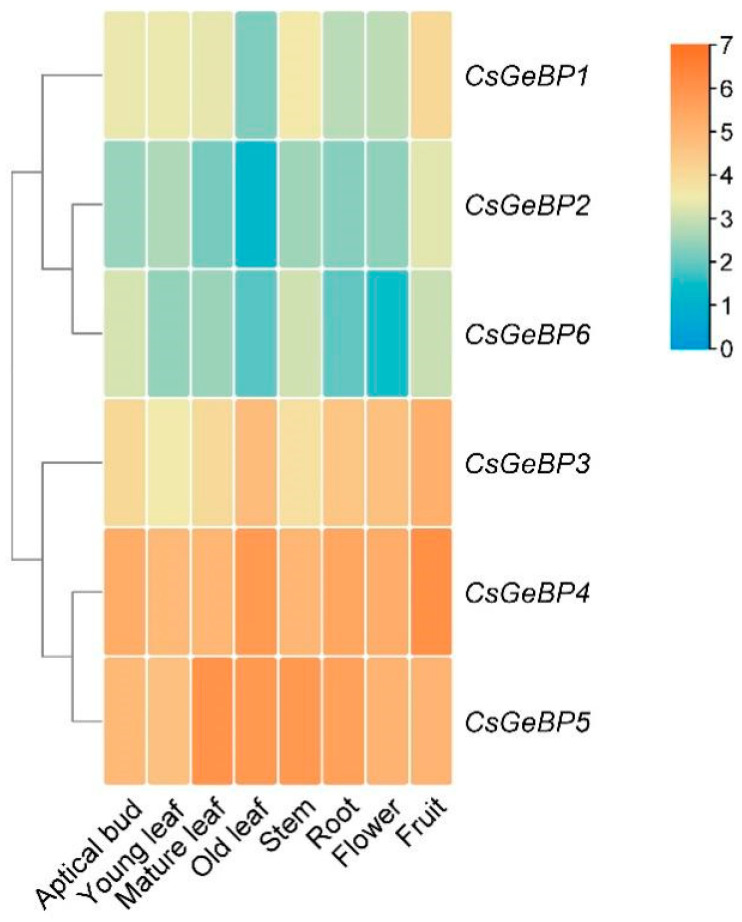
Expression profiles of *CeGeBPs* in different tissues of tea plants. Log2-based fold changes were used to create the heatmap based on the RNA-seq data downloaded from TPIA (http://tpia.teaplant.org, accessed on 13 June 2022). The gene expression level is displayed in different colors on the map, as shown in the bar at the upper right corner.

The expression patterns of the *CsGeBP* genes identified using publicly available RNA-Seq data from eight tissues (apical bud, young, mature, and old leaves, stem root, flower, and fruit) of the tea plant ‘Shuchazao’ were analyzed (Figure 8) [54,55]. *CsGeBP4* and *CsGeBP5* were expressed at higher levels in almost all tissues compared with other gene members (Figure 8). *CsGeBP3* was highly enriched in old leaf, root, flower, and fruit, while the expression levels of *CsGeBP1*, *CsGeBP2*, and *CsGeBP6* were lower in most tested tissues in comparison with other genes (Figure 8). These findings suggested that *CsGeBP3*, *CsGeBP4*, and *CsGeBP5* might play critical regulatory roles during tea plant growth and development. However, the expression patterns of these genes were not well-correlated to the tender degree of leaves, as well as the general distribution patterns of tea trichomes, indicating that the regulatory roles of *CsGeBP* genes in tea trichome formation might be distinct among different tea plant varieties.

**Figure 9:**
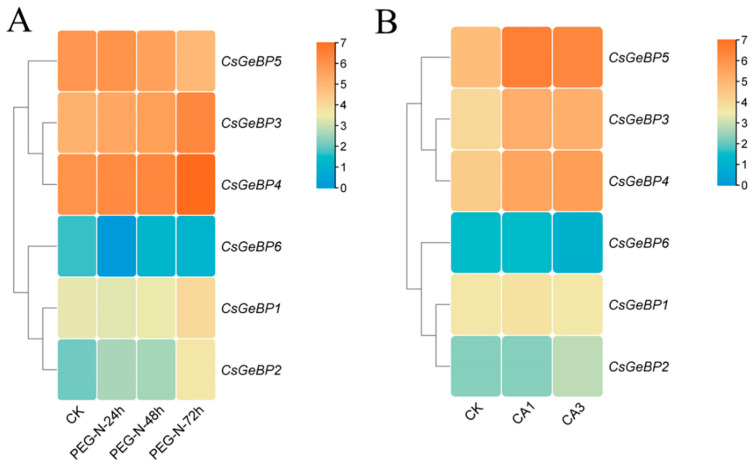
Expression profiles of *CsGeBPs* under drought (**A**) and cold (**B**) stresses. Log2-based fold changes were used to create the heatmap based on the RNA-seq data downloaded from TPIA (http://tpia.teaplant.org, accessed on 13 June 2022). The gene expression levels are displayed in different colors on the map, as shown in the bar at the upper right corner. Drought treatments were conducted by applying 25% polyethylene glycol (PEG) treatment for 0, 24, 48, and 72 h [56]. Cold treatments included non-acclimated (CK), fully acclimated (CA1), and de-acclimated stages (CA3) [57].

To elucidate the expression profiles of the *CsGeBP* genes that are responsive to environmental factors, their expression levels in response to drought and cold treatments were analyzed using the downloaded abiotic stress-responsive transcriptome data (Figure 9) [54,56,57,58]. *CsGeB2*, *CsGeB3*, and *CsGeBP4* showed similar change patterns, being slightly induced in response to drought stress for 72 h, although the expression level of *CsGeB2* was lower than the expression levels of *CsGeB3* and *CsGeBP4* under both control and treatment conditions (Figure 9A). *CsGeB5* displayed a slight decrease after treatment with drought stress for 72 h, while *CsGeB1* and *CsGeBP6* showed no significant changes in response to drought stress and were expressed at low levels in both control and stress-treated plants (Figure 9A). Furthermore, *CsGeB2*, *CsGeB3*, *CsGeB4*, and *CsGeBP5* were all induced by cold stress, while the expression level of *CsGeB2* was lower than the other three genes in both control and stressed plants (Figure 9B). Moreover, the expression levels of *CsGeB1* and *CsGeBP6* were not significantly affected by cold treatments (Figure 9B). These results suggest that *CsGeBP* family members might play critical roles in tea plant defense against environmental stresses.

### 2.7. CsGeBP4 was Highly Related to the High-Dense Trichome Phenotype of Tea Plants

**Figure 10:**
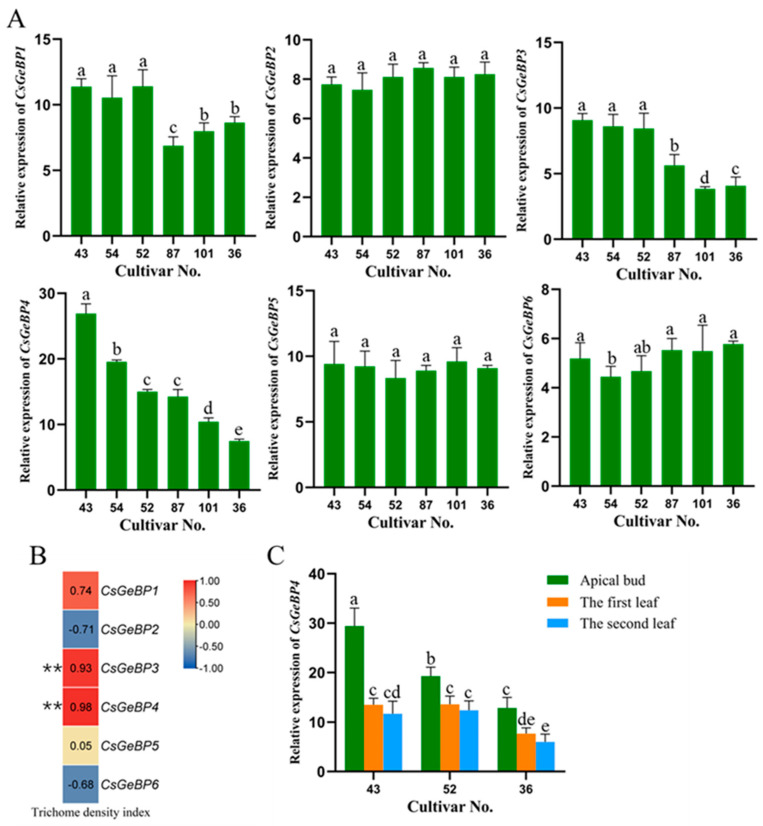
Expression profiles of *CsGeBPs* and their correlation with the tea plant trichome phenotypes. (**A**) Expression levels of *CsGeBPs* in the apical buds of different cultivars of Yunwu Tribute Tea. (**B**) Pearson correlation analysis of the expression of *CsGeBPs* and the trichome density index of tea plants. “**” represents a significantly high degree of correlation (*p* < 0.01). Red indicates a positive, and blue represents a negative correlation. (**C**) The expression level of *CsGeBP4* in the apical buds, the first and the second leaves of different cultivars of Yunwu Tribute Tea. Lowercase letters indicate significant differences among different samples (*p* < 0.05).

To confirm the regulatory roles of *CsGeBPs* in the trichome formation, the expression levels of *CsGeBPs* in the apical buds of different cultivars of Yunwu Tribute Tea were verified using qRT-PCR (Figure 10A). *CsGeBP1*, *CsGeBP3*, and *CsGeBP4* were highly expressed in tea plant cultivars with relatively higher TDIs (Figure 10A and Figure 1B,C). Among them, the expression level of *CsGeBP4* showed the highest correlation with the high-density trichome phenotype (*r* = 0.98, *p* = 0.001) (Figure 10B). We further tested the tissue expression patterns of *CsGeBP4* by using apical buds, the first and the second leaves of three representative cultivars. The transcript level of *CsGeBP4* in each tea plant cultivar was higher on the apical buds, where trichomes are mostly present, than those on the first and second leaves, although the expression level of *CsGeBP4* was similar between the first and the second leaves (Figure 10C). These results strongly indicate that *CsGeBP4* could be a critical regulator involved in trichome formation in tea plants.

### 2.8. Silencing of CsGeBP4 Inhibited Trichome Development in Tea Plants

**Figure 11:**
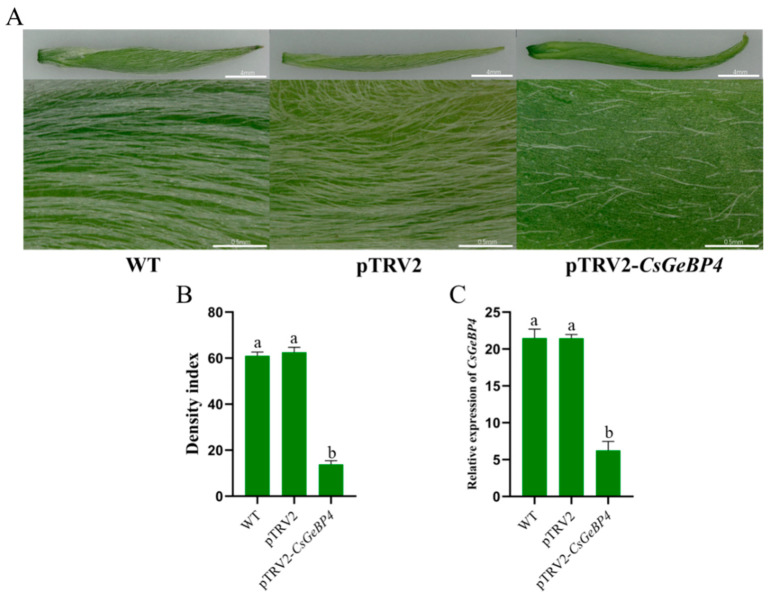
TRV-mediated gene silencing of the *CsGeBP4* in the apical buds of tea plants. Trichome phenotype (**A**), trichome density index (**B**), and the expression level of *CsGeBP4* in the tea cuttings from apical buds of tea plant variety, ‘Fudingdabai’ (**C**). WT, wild-type tea cuttings; pTRV2, tea cuttings infected by pTRV1 + pTRV2 *Agrobacterium*; pTRV2-*CsGeBP4*, tea cuttings infected by pTRV1 + pTRV2-*CsGeBP4 Agrobacterium*. Lowercase letters indicate significant differences among different samples. TRV, tobacco rattle virus; WT, wild type.

The in vivo function of *CsGeBP4* in trichome formation was further verified using the newly developed VIGS technology in the tea plants [40]. The apical buds of *CsGeBP4*-silenced tea plants displayed defective in trichome development, and their TDIs were significantly reduced by 2.3-fold compared with wild-type and control plants (Figure 11A,B). The transcript level of *CsGeBP4* was significantly repressed in *CsGeBP4*-silenced tea plants compared with wild-type and control plants, as verified using qRT-PCR (Figure 11C). These results strongly suggest that *CsGeBP4* was required for tea trichome formation.

## 3. Discussion

Tea plant trichomes contribute to the unique flavor and nutritional quality of tea and protect tea plants from various environmental stresses by producing numerous metabolites, such as catechins, theanine, caffeine, flavonoids, and volatiles [1]. However, the regulatory mechanism underlying trichome initiation, growth, and development in tea plants is not fully understood. By integrating morphological, transcriptomic, bioinformatics, as well as qRT-PCR- and VIGS-mediated gene silencing strategies, we identified and characterized the *CsGeBP* gene family potentially involved in trichome formation and further specified the regulatory role of *CsGeBP4* during trichome growth and development.

### 3.1. Association of CsGeBP Gene Family with High-Dense Trichome Phenotypes in Tea Plants

Tea trichomes are a unicellular, unbranched, and non-glandular type of surface hairs [1,37]. Many studies have demonstrated that fresh buds and tender leaves had higher trichome density than older leaves [1,6,37]. Apart from the impact of tenderness degree, tea plant variety and cultivar also affected tea trichome density [4]. Most modern tea plant cultivars, including both assamica and sinensis types of *C. sinensis*, have a higher density of trichomes than many wild tea relative plants in Section *Thea*, such as *C. tachangensis*, *C. taliensis*, and *C. angustifolia*, most of which have glabrous leaves or low trichome density [1,4,59]. Therefore, densely spaced trichomes on the apical buds and young leaves were considered to be one of the most important domestication traits for better tea flavor and plant development during the millennia-long history of tea plant cultivation. In the present study, a morphological investigation showed that the density index of tea trichomes was continuously distributed among 108 different cultivars of Yunwu Tribute Tea (Figure 1A). The trichome density was much higher in the hairy cultivars in comparison with hairless cultivars (Figure 1B,C), indicating the cultivar-specific trichome phenotypes existed within Yunwu Tribute Tea, and they are considered to be suitable materials for functional studies on tea plant trichome formation.

Tea plant trichome initiation, growth, and development are regulated by numerous genes, especially transcription factors. The previous comparative transcriptomics studies conducted in hairy and hairless tea plants revealed that a wide array of transcription factors display differential expression patterns and are thought to participate in the regulation of tea trichome formation [2,5]. These transcription factors belong to various gene families, mainly AP2/ERF, bHLH, bZIP, C2H2, GRAS, GRF, HB, MYB, NAC, SPL, TCP, Trihelix, WD40, WRKY, etc. [2,5]. Among these, the regulatory functions of transcription factors from MYB, bHLH, and WD40 gene families have been widely studied. Some members of these families can form an MYB-bHLH-WD40 protein complex to activate the downstream regulator genes *GL2*, *CPC*, and other effectors, thereby regulating trichome growth and development [4,5,23]. In this study, a comparative transcriptomics analysis was conducted for two cultivars of Yunwu Tribute Tea. Great differences in trichome density revealed that *CsGeBP4* was among the top 30 up-regulated transcription factor genes with the highest fold changes in expression levels in the hairy tea cultivar compared with the hairless cultivar (Figure 2). The potential relationship between the *GeBP* family and plant trichome formation has been reported in other plants, such as *Arabidopsis* and soybean [42,43,50]. However, the function of *CsGeBPs* in tea trichome growth and development is still elusive. Therefore, further analysis of *CsGeBP* gene family characteristics is necessary for uncovering the regulatory functions in tea plant trichome formation.

### 3.2. Genome-Wide Analysis of GeBP Gene Family and Their Involvement in Tea Plant Growth and Environmental Responses

*GeBP* gene family has been identified and characterized in soybean [50], mango [38], moso bamboo [51], tomato [52], and nine Gramineae crops [45]. However, so far, the systematic analysis of *GeBPs* has not been reported in tea plants. Herein, the genome-wide identification and characterization of *GeBP* family members were performed to provide clues about their potential functions in tea trichome formation. A total of six *CsGeBPs* distributed in four out of fifteen chromosomes were classified into two main phylogenetic groups (Figure 3 and Figure 4). The multi-species phylogenetic analysis showed that the number of *GeBPs* found in the genomes of different plant species varied in each group. *GeBPs* in Group I were expected to be conserved across all four species due to the findings that this group included *GeBPs* from all the tested species (Figure 3). *GeBPs* in Group II, III, and VI exhibited dicot-, *Arabidopsis*-, and rice-specific phylogenetic clustering patterns, indicating that the *GeBP* gene family evolved in multiple directions among these species (Figure 3). The investigation of gene structure features revealed that the number of introns in *CsGeBPs* ranged from zero to one (Figure 5). It was reported that *GeBPs* in soybean also contained no more than one intron [50]. Our results indicate that the gene structure of *GeBPs* was relatively stable and not prone to experience alternative splicing during gene replication. In addition, the analysis of conserved protein motifs revealed that closely related CsGeBP proteins on adjoining branches of the phylogenetic tree had similar motif constituent (Figure 6), which was consistent with the findings in soybean [50] and moso bamboo [51].

The *cis*-regulatory elements in the gene promoters may provide valuable information for further investigating the function of the *CsGeBP* gene family due to their critical roles in the regulation of developmental- and environmental-related gene expression patterns. In this study, the *cis*-regulatory elements of *CsGeBPs* were found to participate in the responses to various environmental factors, including light, drought, low temperature, and anoxic induction (Figure 7A,C). Moreover, the analysis of phytohormone-responsive elements indicated that MeJA, ABA, auxin, and salicylic acid could also impact the expression patterns of *CsGeBPs* (Figure 7B). In addition, the presence of tea-plant-development-related elements in the *CsGeBPs* promoters indicated their potential roles in the regulation of tea plant growth and development (Figure 7D). The tissue-specific and environmental-responsive expression patterns of *CsGeBP* genes revealed using RNA-seq data provided further evidence for these speculations (Figure 8 and Figure 9). *CsGeBP3* and *CsGeBP4* were up-regulated in response to both drought and cold treatments and showed relatively high levels in almost all tested tissues, which could be partially attributed to drought- and cold-responsive *cis*-elements, as well as development-related *cis*-elements in their gene promoters (Figure 7, Figure 8 and Figure 9, Table S2). These results facilitate a further understanding of the regulatory roles of *CsGeBPs* in tea trichome formation and stress responses.

### 3.3. CsGeBP4 Is Required for Tea Plant Trichome Formation

Previous studies reported that an AtGeBP could bind to the *cis*-regulatory elements in the promoter of trichome-formation-related gene *AtGL1* in yeast and in vitro, and was regulated by the meristem establishment gene, *KNAT1* [42,43], suggesting its possible regulatory roles in plant trichome formation. In order to further verify the involvement of *CsGeBPs* in tea plant trichome formation, the association analyses of expression patterns of all *CsGeBP* genes in the tea plant genome with trichome phenotypes among different cultivars of Yunwu Tribute Tea revealed that *CsGeBP4*, with the highest positive correlation efficiency, was strongly related to the high-density trichome phenotype (Figure 10A,B). Moreover, the expression level of *CsGeBP4* was much higher in the apical buds than that in the first and second leaves, which was well-supported by previous findings that apical buds in tea plants had more trichomes than older leaves (Figure 10C) [1,6,37]. These results indicate that *CsGeBP4* might play a critical and potential positive regulatory role in tea trichome growth and development. To verify this speculation, the gene expression of *CsGeBP4* was silenced in the tea plants via the newly developed VIGS strategy [40]. Silencing of *CsGeBP4* led to the repressed gene expression and defect trichome development, as well as the significantly decreased trichome density in the *CsGeBP4*-silenced tea plants, which accurately supported our previous hypothesis and further demonstrated that *CsGeBP4* was required for tea trichome formation. However, the expression of *GmGeBP4* identified in soybean was found to be induced in abnormal trichome soybean with fewer trichomes in comparison to control plants [50], suggesting that the regulatory patterns of *GeBP* genes were distinct across plant species. Therefore, more functional investigations are needed to identify the upstream and downstream genes and/or proteins associated with *GeBPs* and uncover the specific regulatory mechanisms in both tea plants and other plant species.

## 4. Materials and Methods

### 4.1. Plant Materials and Morphological Observations

Yunwu Tribute Tea (*Camellia sinensis* (L.) Kuntze var. niaowangensis Q. H. Chen) plants were grown under standard field conditions at a tea plantation in Yunwu town in Guizhou Province in China (latitude 26°17′ N, longitude 107°03′ E, and altitude 1200 m above mean sea level). Tea plant variety ‘Fudingdabai’ were grown under standard field conditions at the experimental farm of Guizhou Academy of Agricultural Sciences (latitude 26°11′ N, longitude 106°27′ E, and altitude 1185 m above mean sea level, Guiyang, China). Apical buds, first leaves, and second leaves of five plants were sampled as one biological replicate in early spring of 2022. Three biological replicates of different tissues were either used fresh or immediately frozen in liquid nitrogen and stored at −80 °C. The trichome phenotype of apical buds of the selected cultivars was observed using a digital microscope system (VHX-6000, KEYENCE, Osaka, Japan). The density index of trichomes was further analyzed using ImageJ software (https://imagej.en.softonic.com/, accessed on 10 April 2022).

### 4.2. Identification of GeBP Family Genes in the Tea Plant Genome

To identify the GeBP gene family in tea plants, we used *Arabidopsis* AtGeBP amino acid sequences to search against tea proteomes downloaded from Tea Plant Information Archive (TPIA) (http://tpia.teaplant.org/, accessed on 13 June 2022) using a Basic Local Alignment Search Tool (BLAST-P) (E-value < 10^−5^) and deleting redundant sequences. Candidate CsGeBP protein sequences were submitted to the online bioinformatics tool, the National Center of Biotechnology Information Conserved Domain-Search Tool (NCBI CD-Search Tool) (https://www.ncbi.nlm.nih.gov/Structure/index.shtml, accessed on 13 June 2022) (E-value < 10^−2^) to verify their conserved domains. The number of amino acid residues, gene structure (exon/intron arrangement), and start-to-end position of each gene in the genome were analyzed based on the annotated information downloaded from the tea plant genome database. Physical parameters of each gene product, such as molecular weight and isoelectric point, were predicted using ExPASy (http://web.expasy.org/compute_pi/, accessed on 15 June 2022). The subcellular localization of each CsGeBP was predicted by WoLF PSORT (https://wolfpsort.hgc.jp/, accessed on 15 June 2022).

### 4.3. Phylogenetic Analysis of GeBPs among Different Plant Species

An evolutionary relationships analysis of GeBP proteins from tea plants, *Arabidopsis*, rice, and soybean was carried out using MEGA X (Mega Limited, Auckland, New Zealand) [60] based on the neighbor-joining method [61]. Bootstrap test replicates were set to 1000 times [62]. The phylogenetic tree was displayed using FigTree software (version 1.4.2). Protein sequences of *Arabidopsis*, rice, and soybean were obtained from The *Arabidopsis* Information Resource (TAIR) (https://www.arabidopsis.org/, accessed on 13 June 2022), Rice Genome Annotation Project (RGAP) (http://rice.plantbiology.msu.edu/index.shtml, accessed on 13 June 2022), and SoyBase (http://www.soybase.org/, accessed on 13 June 2022), respectively.

### 4.4. Chromosomal Distribution, Gene Structure, and Conserved Motif Analysis

The chromosomal localization information (including chromosomal distribution, length, as well as the start and end positions) of *CsGeBP* genes was downloaded from TPIA (http://tpia.teaplant.org/, accessed on 13 June 2022) and visualized by TBtools. The exon–intron structures of the CsGeBP genes based on the genome annotations were visualized using TBtools software. The conserved protein motifs of CsGeBP proteins were analyzed through the Multiple Em for Motif Elicitation (MEME) program (http://meme-suite.org/, accessed on 14 June 2022) and visualized using TBtools.

### 4.5. Identification of Putative Cis-Regulatory Elements in the Promoters of CsGeBPs

The promoter sequences (2000 bp upstream of the start codon) of all *CsGeBP* genes were obtained from TPIA (http://tpia.teaplant.org/, accessed on 13 June 2022), analyzed by using the PlantCARE (http://bioinformatics.psb.ugent.be/webtools/plantcare/html/, accessed on 14 June 2022), and visualized by TBtools.

### 4.6. Expression Analysis of CsGeBPs in Different Tissues and in Response to Environmental Stresses

The transcriptome data (TPM value) of *CsGeBPs* in eight tissues (apical bud, young leaf, mature leaf, old leaf, stem, root, flower, and fruit) of tea plant cultivar ‘Shuchazao’ and transcriptome data in ‘Longjing43′ and ‘Tieguanyin’ in response to drought and cold stresses, respectively, were downloaded from TPIA (http://tpia.teaplant.org, accessed on 13 June 2022). The log2-based fold changes were used to create heatmaps by TBtools.

### 4.7. RNA Extraction and qRT-PCR Analysis

Primers for *CsGeBP* gene cloning were designed using the online program Integrated DNA Technologies (IDT) (https://sg.idtdna.com/pages, accessed on 5 July 2022) (Supplementary Table S3), and the primer sequences were synthesized by Beijing Qingke Biotechnology limited company. Total RNA was extracted from apical buds, and the second leaves were extracted using a cetyltrimethylammonium bromide (CTAB) method [63]. The RNA was reverse transcribed into first-strand cDNA using the PrimeScript^TM^ II first-strand cDNA Synthesis Kit (Solarbio Technology, Beijing, China). The cDNA was subsequently employed as a template for qRT-PCR analysis using SYBR Green qPCR Mix (Genenode, Wuhan, China) reagent. Each reaction system contained 10 μL SYBR Green qPCR Mix, 0.8 μL primers, 1.5 μL cDNA template, 7.7 μL H_2_O. The reaction process is as follows: 95 °C for 3 min; 40 cycles of 95 °C for 10 s and 60 °C for 20 s; 72 °C for 30 s. *CsGAPDH* was used as the reference gene. The relative expression level was calculated using the 2^−∆∆CT^ method [64].

### 4.8. Differentially Expressed Gene Analysis by Transcriptome Sequencing

Total RNA was extracted from apical buds of two cultivars (No. 43 and No. 36) of Yunwu Tribute Tea using the RNeasy Plus Mini kit (Qiagen, Valencia, CA, USA). Three independent biological replicates were used for RNA sequencing. RNA integrity was evaluated using the Agilent 2100 Bioanalyzer (Agilent Technologies, Palo Alto, CA). The samples with RNA integrity number (RIN) ≥ 7 were submitted to enrich mRNA and construct cDNA libraries using TruSeq Stranded mRNA LTSample Prep Kit (Illumina, San Diego, USA) according to the manufacturer’s instructions. The libraries were sequenced using the Illumina HiSeq™ 2000 platform (Illumina, San Diego, USA). In order to obtain high-quality clean reads, adaptor sequences, empty reads, and low-quality bases (Q < 30) were removed. The resulting clean reads were subsequently used for transcriptome de novo assembly by mapping to the tea plant reference genome (http://tpia.teaplant.org/, accessed on 20 May 2022) [54,58]. Fragments per kilobase of transcript per million (FPKM) of each gene and read counts value of each transcript (protein_coding) were calculated using bowtie2 and eXpress. The differential expressions of genes between the two tea plant cultivars were analyzed using the DESeq (2012) R package. The FPKM values between two cultivars were compared using a threshold of FDR < 0.001 and |log2ratio| > 1 to investigate differentially expressed genes.

### 4.9. VIGS-Based Gene Silencing in Tea Plants

Gene silencing of *CsGeBP4* using VIGS technology was performed in the tea plant variety ‘Fudingdabai’ as described previously [40]. Briefly, a 292 bp fragment of *CsGeBP4* used for VIGS was assembled into the pTRV2 virus vector to construct the pTRV2-*CsGeBP4* vector. Then, pTRV1, pTRV2, and pTRV2-*CsGeBP4* were transformed into *Agrobacterium tumefaciens* strain GV3101, respectively. After cultivation and resuspension, *Agrobacterium* harboring pTRV1 were mixed with those harboring pTRV2 or pTRV2-*CsGeBP4*, respectively, and they were both infiltrated into tea plant cuttings via vacuum infiltration, respectively. The inoculated tea cuttings were kept in the dark for three days and then grown in a greenhouse at 25 °C under a 16 h/8 h light/dark cycle.

### 4.10. Statistical Analysis

All results are presented as means ± standard deviation of at least three biological replicates. The data were subjected to one-way analysis of variance using SPSS software (version 26.0). *p*-values less than 0.05 were considered statistically significant.

## 5. Conclusions

In this study, we reported the association of *CsGeBP* family members with high-density trichome phenotypes in tea plants. Our genome-wide analysis of the *CsGeBP* gene family in tea plants and the expression patterns of *CsGeBPs* in multiple tissues in response to environmental factors revealed valuable information for understanding the potential biological roles of *CsGeBPs* in tea trichome formation. More importantly, we demonstrated that *CsGeBP4* was required for tea trichome formation, potentially for positive regulation. This study provides new insights into the understanding of tea trichome formation and lays a foundation for future research to improve the flavor and quality of tea products and breed stress-tolerant tea plant cultivars.

## Figures and Tables

**Table 1:** Physical and molecular properties of CsGeBPs identified in tea plant genome.

Gene ID	Gene Name	Chromosome No.	Length (bp)	Intron	Exon	Amino Acid (aa)	Molecular Weight (Da)	Isoelectric Point	Subcellular Localization
CSS0047893.1	CsGeBP1	Chr1	1363	0	1	370	40,951.80	8.28	Nucleus
CSS0008596.1	CsGeBP2	Chr1	673	1	2	221	24,638.69	9.85	Nucleus
CSS0012610.1	CsGeBP3	Chr6	1675	1	2	384	42,104.87	4.63	Nucleus
CSS0010019.1	CsGeBP4	Chr6	2624	1	2	410	45,031.43	4.65	Nucleus
CSS0022087.1	CsGeBP5	Chr7	881	1	2	283	31,931.65	9.40	Nucleus
CSS0046322.1	CsGeBP6	Chr14	1325	0	1	330	36,379.28	5.41	Nucleus

## Data Availability

The data presented in this study are available on request from the corresponding author.

## Supplementary Materials

The following supporting information can be downloaded at: https://www.mdpi.com/article/10.3390/ijms24065207/s1.
